# LOXL3 Function Beyond Amino Oxidase and Role in Pathologies, Including Cancer

**DOI:** 10.3390/ijms20143587

**Published:** 2019-07-23

**Authors:** Talita de S. Laurentino, Roseli da S. Soares, Suely K. N. Marie, Sueli M. Oba-Shinjo

**Affiliations:** Laboratory of Molecular and Cellular Biology (LIM 15), Department of Neurology, Faculdade de Medicina FMUSP, Universidade de Sao Paulo, Sao Paulo, SP 01246-903, Brazil

**Keywords:** LOXL3, lysyl oxidase, collagen, development, disease, cancer

## Abstract

Lysyl oxidase like 3 (LOXL3) is a copper-dependent amine oxidase responsible for the crosslinking of collagen and elastin in the extracellular matrix. LOXL3 belongs to a family including other members: LOX, LOXL1, LOXL2, and LOXL4. Autosomal recessive mutations are rare and described in patients with Stickler syndrome, early-onset myopia and non-syndromic cleft palate. Along with an essential function in embryonic development, multiple biological functions have been attributed to LOXL3 in various pathologies related to amino oxidase activity. Additionally, various novel roles have been described for LOXL3, such as the oxidation of fibronectin in myotendinous junction formation, and of deacetylation and deacetylimination activities of STAT3 to control of inflammatory response. In tumors, three distinct roles were described: (1) LOXL3 interacts with SNAIL and contributes to proliferation and metastasis by inducing epithelial-mesenchymal transition in pancreatic ductal adenocarcinoma cells; (2) LOXL3 is localized predominantly in the nucleus associated with invasion and poor gastric cancer prognosis; (3) LOXL3 interacts with proteins involved in DNA stability and mitosis completion, contributing to melanoma progression and sustained proliferation. Here we review the structure, function and activity of LOXL3 in normal and pathological conditions and discuss the potential of LOXL3 as a therapeutic target in various diseases.

## 1. Introduction

**Figure 1:**
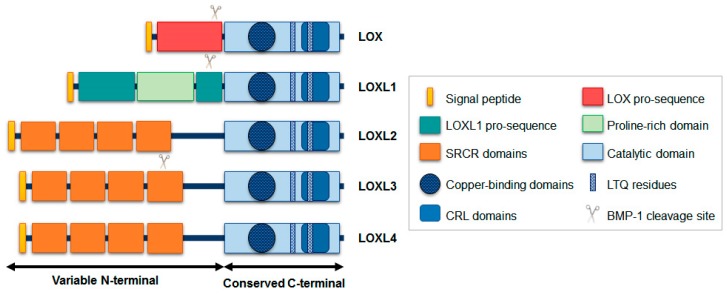
Schematic diagrams of lysyl oxidase family members. The C-terminal is conserved among all five members, and contains the catalytic portion with copper-binding domain, lysyl-tyrosyl quinone (LTQ) residues, and cytokine receptor-like (CRL) domain. On the other hand, the N-terminal is variable: LOX and LOX1 has propeptide sequences, while LOXL2, LOXL3, and LOXL4 have four scavenger receptor cysteine-rich (SRCR) domains. LOX, LOXL1, and LOXL3 have putative sites for BMP-1 cleavage.

The lysyl oxidase (LOX) family is composed of five members: LOX, LOXL1, LOXL2, LOXL3, and LOXL4. All members are copper-dependent amine oxidases responsible for the covalent crosslinking of soluble collagen and elastin chains into insoluble forms. They contribute to extracellular matrix (ECM) stiffness and stabilization. Although the C-terminal regions of the members of this family are conserved, the N-terminal portions are variable. Accordingly, this family is divided into two groups based on the N-terminal similarities. LOX and LOXL1 have N-terminals with pro-sequences, which confer their secretion as inactive pro-enzymes, whereas LOXL2, LOXL3, and LOXL4 contain scavenger receptor cysteine-rich (SRCR) domains, as previously reviewed [1,2,3]. The structural similarities and differences among all LOX family members are presented in Figure 1. The amino acid sequence similarity is higher between LOXL3 and LOXL2 with 69% homology, whereas between LOXL3 and the other members, LOX, LOXL1, and LOXL4 homology is around 50% [4,5].

LOXL3 is the member of the LOX family on which fewer studies have been conducted. Initially, LOXL3 activity as an amino oxidase was based on the homology with the other members of the family. Recently, more studies about LOXL3 have been published, and a variety of new roles have been attributed to LOXL3. Here, we review the structure, function, and activity of LOXL3 development and various pathologies.

## 2. LOXL3: Gene and Protein Structure

Human *LOXL3* (ENSG00000115318) was identified in 2001 by three independent groups, through human EST (expressed sequence tag) database searches of sequence similarities to gene coding for other LOX members [5] or homology to mouse *Lor2* [6]. The isolated cDNA predicted a protein with similar domains of previously identified *LOX*, *LOXL1*, and *LOXL2*.

**Figure 2:**
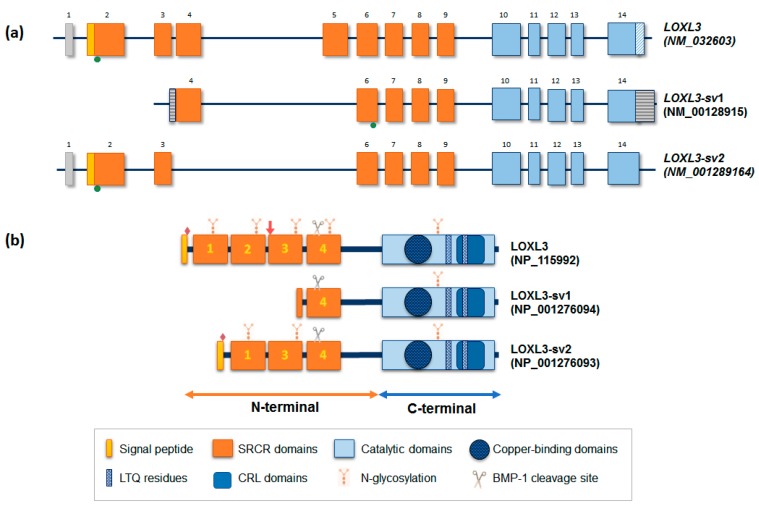
*LOXL3* mRNA and protein variants. (**a**) Schematic diagrams of the intron-exon structure of the three transcript variants of human *LOXL3*, with exons represented by boxes, and intron as bold line. The longest full-length transcript of *LOXL3* has 14 exons, with a 5’UTR (gray box) and 3’UTR (hatched blue box). The transcript *LOXL3-sv1* lacks exons 1, 2, 3, and 5, and has different 5’UTR and 3’UTR regions (dashed boxes). The transcript variant *LOXL3-sv2* differs from the full-length transcript by lacking exons 4 and 5. The orange color represents the exons that code for the N-terminal region, the yellow color represents the region that codes for the signal peptide sequence, and blue color represents the exons that code for the C-terminal region. Green dots represent the start translation codon location. (**b**) Schematic diagrams of LOXL3 protein variant structures, with signal peptide and scavenger receptor cysteine-rich (SRCR) domains in the N-terminal region, and catalytic domain (blue) in the C-terminal region. Red diamonds indicate the putative O-glycosylation sites; red arrows indicate bipartite nuclear localization signal. All protein variants has a common catalytic domain with cooper binding region, lysyl-tyrosyl quinone (LTQ) residue, and cytokine receptor-like (CRL) domain.

*LOXL3* is mapped to chromosome 2p13.3, presenting 23,462 nucleotides, 14 exons, and the full-length cDNA with 3,121 bp (Figure 2a). The 5’-flanking region of *LOXL3* in exon 1, corresponding to the promoter region, has no typical TATA or CAAT boxes, but presents potential transcription factor binding sites for STAT3, STAT6, SRF MIBP/RFX1, SP1, NF1, NRSF, CRE binding protein 1, PAX6 paired domain, IRF-related protein, GATA binding factor 1, NF-κB, GAGA box, c-Rel sites, and AP-2 [7].

The open reading frame of the longest full-length transcript variant encodes a 753 amino acid protein with a molecular mass of 80.3 kDa. The C-terminal portion comprises the catalytic domain; it has a copper-binding motif, lysyl-tyrosyl-quinone (LTQ) cofactor residues, required for protein conformation and catalytic activity, and a cytokine receptor-like (CRL) domain [5,6,8]. This catalytic region is conserved in other LOX-like proteins, particularly the regions coded by exons 10, 11, and 12. The N-terminal region of LOXL3, corresponding to the exons 2 to 9, contains four SRCR domains, and a putative extracellular signal peptide cleavage site, between residues 25 and 26 (Figure 2) [5]. Based on its predicted structure, LOXL3 can be secreted and processed in the extracellular space by bone morphogenetic protein 1 (BMP-1) at a cleavage site GDD (between residues 446–448) [6], in a similar process to that LOX and LOXL1 (Figure 1) [9]. The cleaved product has a predicted size of 35 kDa, with 306 amino acids [1,6,10].

Additionally, LOXL3 can be glycosylated. LOXL3 presents three putative sites for O-glycosylation (Ser-26, Ser-28 and Ser-30) just after the signal peptide sequence cleavage site, which are not found in other LOX-like members, and five sites for N-glycosylation along the protein (Figure 2b) [5]. Therefore, glycosylation of any of these sites can explain the discrepancy in the observed secreted protein size. In fact, recombinant LOXL3 was recovered in concentrated medium from fibrosarcoma cells (HT-1080), with a size of 97 kDa, a larger molecular weight than expected probably due to glycosylation [5].

Additionally, LOXL3 can also be translocated to the nucleus due to a bipartite nuclear localization signal (residues 293–311), composed of two clusters of basic amino acids [5]. Additionally, there is a putative nuclear exporting signal in the N-terminal region which overlaps with the signal peptide [11].

Additionally, two isoforms of *LOXL3*, termed *LOXL3-sv1* and *LOXL3-sv2*, were identified during the search for *LOXL3* homologues in the human EST database [7,12]. *LOXL3-sv1* is the shortest transcript and lacks exons 1, 2, 3, and 5. In addition, the 5’-flanking region of exon 4 contains additional 80 bp as an alternative promoter, and the 3’-end flanking region of exon 14 contains an additional sequence of 561 bp. In contrast to the full-length LOXL3, the *LOXL3-sv1* promoter has a TATA box, and potential binding sites for different transcription factors as for p53, GATA-binding factor 3, STAT5, Pit1, Ras-responsive element binding protein 1, Neurogenin 1 and 3, AP-1, PAX 2/5/8, YY1, and NUDR. Translation of this variant starts at exon 6 and encodes a predicted polypeptide of 392 amino acids with 44 kDa of molecular weight. Despite lacking the first three SRCR domains, LOXL3-sv1 conserves the C-terminal and the amino oxidase activity. Similar to LOXL3, LOXL3-sv1 might also be a target for BMP-1 and has two putative N-glycosylation sites (Figure 2) [7]. *LOXL3-sv2* variant, described more recently, contains 12 exons and lacks the sequences corresponding to exons 4 and 5 of *LOXL3*, without alteration of the open-reading frame. The missing sequences in this alternative splicing encode for SRCR domain 2. The *LOXL3-sv2* transcript variant encodes a 608 amino acid protein with a calculated molecular mass of 67.4 kDa, with putative O-glycosylation and N-glycosylation sites and as BMP-1 cleavage site, as shown in Figure 2 [12]. Although this variant maintains the SRCR3 domain, the bipartite nuclear localization sequence is lost.

## 3. Localization

### 3.1. Tissue-Specific Expression of LOXL3

**Figure 3:**
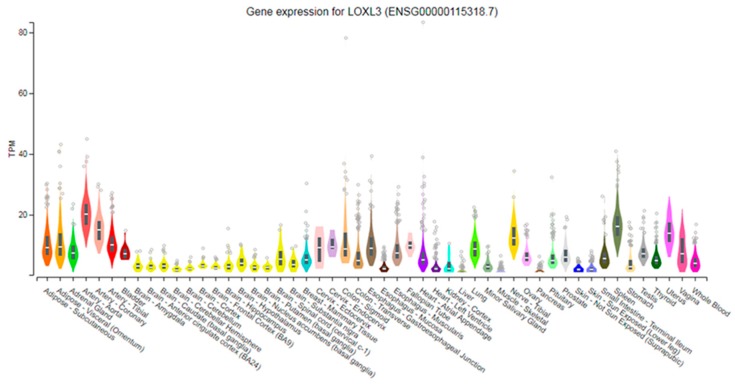
*LOXL3* expression analysis in 51 normal tissues. Gene expression was determined by Genotype-Tissue Expression RNA-Seq database using GTEx Browser (http://www.gtexportal.org/home/), Analysis Release V7. Expression values are shown in transcripts per million (TPM). Violin plots are represented by median and 25th and 75th percentiles. Outliers represented by points are above or below 1.5 times the interquartile range.

Northern blot analysis of *LOXL3* was performed in various normal human tissues, and the band of approximately 3.1–3.2 kb, consistent with the length of the *LOXL3* cDNA sequence, was identified as *LOXL3* mRNA [5,6]. The highest expression levels of *LOXL3* transcript were detected in the heart, spleen, uterus, medulla, spinal cord, and ovary [5,6]. An analysis of *LOXL3* expression levels in a database of normal RNA-human tissues from a large cohort of Genotype-Tissue Expression (GTEx) using the GTEx Browser (http://www.gtexportal.org/home/) was performed and is presented in Figure 3 [13,14,15]. *LOXL3* expression is high in the heart, spleen, and uterus as previously observed by Northern blot analyses. Additionally, *LOXL3* expression is high in the lung, aorta, and coronary arteries (Figure 3), and it has also been described in corneal layers, corneal trabecular meshwork, ocular limbus, and conjunctiva [16].

High *LOXL3-sv1* expressions were detected in the liver, pancreas, spleen, and thymus [7]. High *LOXL3-sv2* expressions were observed in the spleen, testis, ovary, placenta and small intestine [12]. The meaning of these differential expressions of *LOXL3* transcript variants in normal tissues still needs to be elucidated.

### 3.2. Subcellular Localization

Originally, the cellular localization of LOXL3 was described in cytoplasm and extracellular space, as protein secreted into the culture medium by fibrosarcoma cells (HT-1080 cells) [5]. Other studies have shown that LOXL3 was also secreted by cardiomyocytes, and played a role in collagen crosslinking of the ECM [7]. Surprisingly, LOXL3 oxidative effect on the ECM fibronectin of the myotendinous junction has also been described in a mouse model [17]. In the cytoplasm, LOXL3 was observed at perinuclear localization in HeLa cells overexpressing LOXL3, in melanoma cells, and in Madin–Darby canine kidney cells [18,19]. Intranuclear LOXL3 has also been reported in HeLa cells and in mouse spleen cells. In addition, concomitant cytoplasm and nuclear localization was described in gastric cancer [20]. Interestingly, LOXL3 nuclear localization was further confirmed by the demonstration of its interaction with human telomerase reverse transcriptase (hTERT) through yeast two-hybrid and immunoprecipitation assays [21]. The cellular distribution of LOXL3 variants still needs to be completed, as the localization of the LOXL3-sv1 isoform in cytoplasm and extracellular space has been reported only in one study [7]. No studies were found concerning LOXL3-sv2 localization.

### 3.3. Amine Oxidase Activity of LOXL3

**Figure 4:**
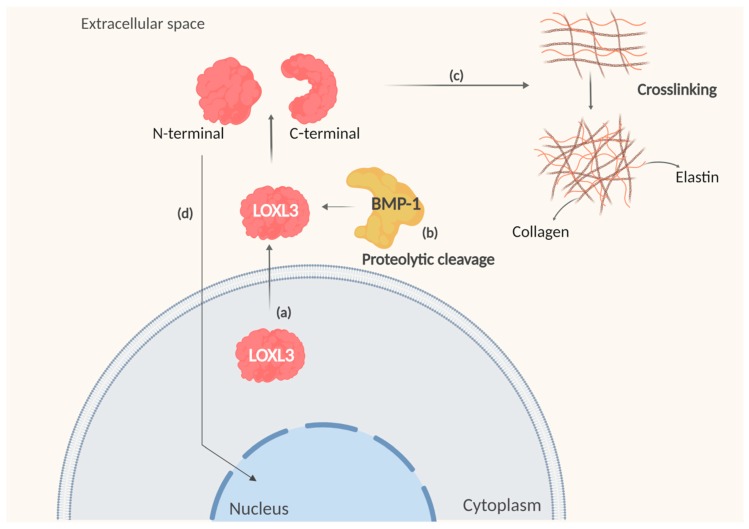
LOXL3 amine oxidase activity. (**a**) LOXL3 protein is synthetized and secreted to extracellular space. (**b**) LOXL3, cleaved by BMP-1 has the C-terminal separated from the N-terminal domain. (**c**) The C-terminal region has the amine oxidase activity and promotes the collagen and elastin crosslinking. LOXL3 showed higher specificity to collagen type I, IV, VIII, and X. (**d**) The N-terminal portion can be translocated to the nucleus. Created with BioRender.

LOXL3 has a putative cleavage sequence site at the fourth SRCR domain (Figure 2b) for BMP-1 [6]. This process occurs in the extracellular space, and generates a catalytically active amino oxidase with a predicted molecular mass of 35 kDa of the C-terminal. However, the cleaved protein from colon tissue by Western blot analysis presented a molecular mass of 40 kDa, possibly due to post-translational modification such as glycosylation. In amine oxidase assay, either LOXL3 or variants showed amine oxidase activity toward different types of collagen (types I, II, III, IV, VI, VIII and X), as well as toward elastin [7] (Figure 4). LOXL3 presents higher activity toward collagen VIII, and LOXL3-sv1 to collagen I, IV, and X [7]. LOXL3-sv2 also showed amine oxidase activity toward collagen type I [12]. LOXL3 amino oxidase activity was inhibited by β-aminopropionitrile (β-APN), a potent and irreversible inhibitor of amino oxidase, which inhibits the formation of crosslinks in vivo [7,12].

## 4. LOXL3 Roles in Development and Diseases

### 4.1. LOXL3 in Craniofacial-Ocular System

**Figure 5:**
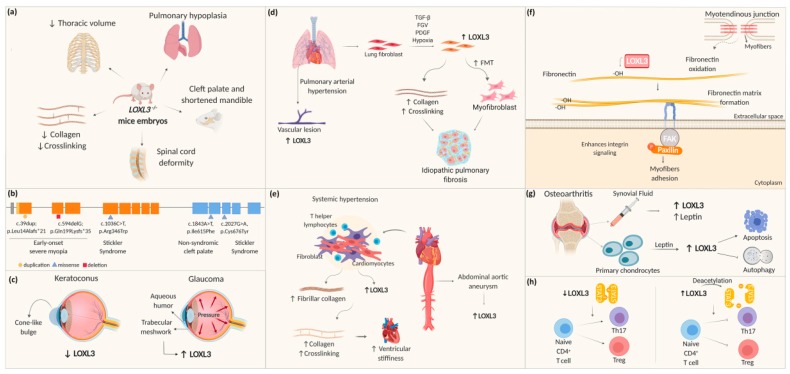
Role of LOXL3 in development and diseases. (**a**) Development: Loxl3-deficitent mouse model demonstrated the critical role of LOXL3 in spinal cord, craniofacial and pulmonary development. (**b**) Mutations: *LOXL3* is mutated in early-onset myopia, Stickler syndrome, and non-syndromic cleft palate patients. (**c**) Ocular system: LOXL3 is downregulated in keratoconus disease and upregulated in trabecular meshwork cells. (**d**) Pulmonary system: LOXL3 expression and activity are upregulated by different growth factors and hypoxia by idiopathic pulmonary hypertension lung fibroblasts. This contributes to high collagen expression and crosslinking and to trans-differentiation of fibroblasts to myoblasts, named fibroblast-to-myofibroblast transition (FMT), suggesting an important role of LOXL3 in lung fibrosis. Additionally, LOXL3 is upregulated in lung vascular lesions of patients with pulmonary arterial hypertension. (**e**) Cardiovascular system: LOXL3 is upregulated in cardiac ECM remodeling in systemic hypertension and in abdominal aortic aneurism. Additionally, cardiac fibroblasts and cardiomyocytes, influenced by T helper lymphocytes, express high levels of LOXL3 resulting in collagen crosslinking. (**f**) Myotendinous system: LOXL3 hydroxylates fibronectin, which activates intracellular integrin signaling, and correct adhesion of myofibers to muscle-tendon junction during development. (**g**) Osteoarticular system: LOXL3 and leptin are upregulated in osteoarthritis cartilage, as well as in synovial fluid of osteoarthritis patients. Osteoarthritis cartilage-derived chondrocytes express high levels of LOXL3 when stimulated by leptin, resulting in induction of autophagy and inhibition of apoptosis. (**h**) Immune system: LOXL3 deacetylates STAT3 and disrupts STAT3 dimerization, inhibiting its transcriptional activity. Consequently, CD4^+^ T cells do not differentiate to Th17 and Treg, suggesting a physiologic role of LOXL3 in immune regulation. Created with BioRender.

The importance and involvement of LOXL3 in development was first suggested in a zebrafish model, in which the lack of *Loxl3b*, the orthologue of mammalian *LOXL3*, caused craniofacial defects [22]. These findings were confirmed in Loxl3-deficient mice (Loxl3^−/−^), which exhibited severe craniofacial defects (cleft palate and shortened mandible) and spinal cord deformities, with a decrease of collagen crosslinks, and early lethality (Figure 5a) [23]. Additionally, Loxl3^−/−^ homozygous mice corresponded to less than 10% of weaned mice, confirming the importance of Loxl3 in embryo viability [11,17].

Interestingly, the features of Loxl3 deficient mouse models overlap with a human collagenopathy, called Stickler syndrome, an autosomal dominant disease caused most frequently by *COL2A1* and *COL11A1* mutations, characterized by high myopia and cleft palate. However, more recently, a homozygous missense variant in *LOXL3* (c.2027G>A, p.Cys676Tyr) was identified in a family with two affected siblings, presenting micrognathia, cleft palate, and severe myopia [24]. No mutations in collagen genes were detected in either sibling. Additionally, a family with SS phenotype was reported as harboring a homozygous novel *LOXL3* mutation (c.1036C>T, p.Arg346Trp), in which two affected members, the father and a child, presented myopia and retinopathy [25]. Moreover, a missense variant of *LOXL3* (c.1843A>T, p.Ile615Phe) was associated with non-syndromic cleft palate, due to lack of catalytic activity of LOXL3 enzyme, with impaired collagen fiber assembly in palatal mesenchyme. Additionally, individuals presenting the Phe/Phe genotype presented a higher risk of non-syndromic cleft palate [26]. LOXL3, like the other members of lysyl oxidases, was also observed in all layers of corneas, as well as in the limbus and conjunctiva. Early-onset severe myopia was associated with homozygous null mutation on *LOXL3* (c.39dup, p.Leu14Alafs*21) and heterozygous combination of this mutation with another frameshift mutation (c.594delG, p.Gln199Lysfs*35) [27]. Gene localization of the five *LOXL3* mutations associated with diseases is presented in Figure 5b.

In keratoconus, a disease characterized by irregular astigmatism resulting in significant visual impairment, LOXL3 expression is downregulated, as well as LOXL2 and LOXL4 expression, suggesting that LOX members may be involved in keratoconus pathogenesis (Figure 5c) [16]. Moreover, the LOX family members, including LOXL3, might also play a role in glaucoma. Lysyl oxidases expression and activity are induced by TGF-β1, TGF-β2, and TGF-β3 via Smad, and non-Smad (JNK and AP-1) signaling pathways in trabecular meshwork aminopropionitrilcells [28]. As TGF-β2 levels are elevated in the aqueous humor and trabecular meshwork cells, lysyl oxidases activity can enhance ECM deposition and trabecular meshwork stiffness, contributing to increased ocular hypertension (Figure 5c) [28].

### 4.2. LOXL3 in Pulmonary and Cardiovascular Systems

Loxl3^−/−^ mice also presented immature lung development, with reduced thoracic cavities and pulmonary hypoplasia (Figure 5a) [29]. Additionally, LOXL3 has been involved in idiopathic pulmonary fibrosis (IPF), a chronic disease characterized by uncontrolled and excessive accumulation of ECM and consequent severe lung dysfunction (Figure 5d). Upregulated LOXL3 expression was detected in IPF tissue compared to control lung tissue [30], more specifically in the cilia bronchial epithelium [31,32]. Increased LOXL3 amino oxidase activity promoting enhanced collagen crosslink may contribute to lung tissue stiffness. The abnormal ECM stimulates fibroblast growth, which in turn may aggravate lung fibrosis [33]. In fact, upregulated lysyl oxidases, including LOXL3, were observed in normal human lung fibroblasts in response to various growth factors (TGF-β, FGF, and PDGF) and hypoxia. These pro-fibrotic stimuli induced fibroblasts-to-myofibroblasts transition (FMT), corroborating the important contribution of LOXL3 in fibrotic lung disease [31]. Nonetheless, inhibition of LOXL3, LOX or other LOX-like proteins has not been efficient for the treatment of IPF.

Another involvement of LOXL3 in pulmonary disease was described in idiopathic pulmonary arterial hypertension. All lysyl oxidases expression levels were elevated in the lungs of those patients, but only LOXL2 and LOXL3 expression was elevated in vascular lesions (Figure 5d) [34].

In abdominal aortic aneurysm, LOXL3 was described as upregulated in the aortic wall of an animal model of vascular sclerotic lesion induced by high fat diet and vasoconstrictor treatment. Also, the use of LOX family inhibitor β-APN resulted in an increase of lesions, suggesting the critical role of LOXL3 in the integrity of the vascular wall [35].

In systemic hypertension, high LOXL3 expression was observed in cardiac fibroblasts and cardiomyocytes, leading to increased fibrillary collagen crosslinking, and cardiac ECM remodeling, which caused ventricular stiffness and cardiac diastolic dysfunction. Interestingly, such LOXL3 response to hypertension was strongly influenced by TH1 lymphocytes, suggesting a differential LOXL3 induction according to immune background (Figure 5e) [36,37].

### 4.3. LOXL3 in Myotendinous and Osteoarticular System

Another function of LOXL3 during development was described in myotendinous junction formation. In mouse development, Loxl3 is expressed in the myotendinous region, and directly oxidizes fibronectin, a ligand of integrin. This interaction activates the integrin signaling pathway, with paxillin phosphorylation, which ensures the correct regulation of ECM scaffold organization and anchoring of myofibers to the muscle-tendon junction (Figure 5f) [17].

LOXL3 involvement was also described in osteoarthritis (Figure 5g). *LOXL3* was one of the genes upregulated in an affected cartilage microarray study of osteoarthritis [38]. In another study, Huang et al. confirmed this finding and found LOX3 in the synovial fluid of patients with osteoarthritis, which positively correlated with leptin levels [39]. A rat model of osteoarthritis induced by anterior cruciate ligament transection further confirmed the higher expression of Loxl3 in affected cartilage. Upregulation of Loxl3 with leptin treatment of primary chondrocytes from affected rat knees with increased apoptosis and concomitant suppression of autophagy corroborated LOXL3 involvement in osteoarthritis and point to it as a potential therapeutic target in this disease [39].

### 4.4. LOXL3 and Immune System

Novel enzymatic activities of LOXL3 in terms of removal of acetyl and acetylamino groups of STAT3 were also reported [11]. These new LOXL3 deacetylation and deacetylimination roles were attributed to the conserved 1–3 SRCR domains of the N-terminal portion of the protein. LOXL3 colocalizes with STAT3 in the nuclei, exerts its dual catalytic activities, and disrupts STAT3 dimerization. Consequently, STAT3 transcription activity is interrupted, restricting cell proliferation and differentiation. Cells with LOXL3 knockdown presented STAT3 constitutive acetylation and increased expression of gene coding for proteins involved in the cell cycle and pluripotency. A physiologic role of LOXL3 in immune regulation was demonstrated by observing that Stat3 is constitutively acetylated in *Loxl3* knockout mice, with consequent reduced CD4^+^ T cell differentiation to Th17 and Treg (Figure 5h). Therefore, Loxl3 negatively regulated inflammatory Th17 and Treg differentiation, thus contributing to the control of inflammatory responses [11].

### 4.5. LOXL3 and Cancer

**Figure 6:**
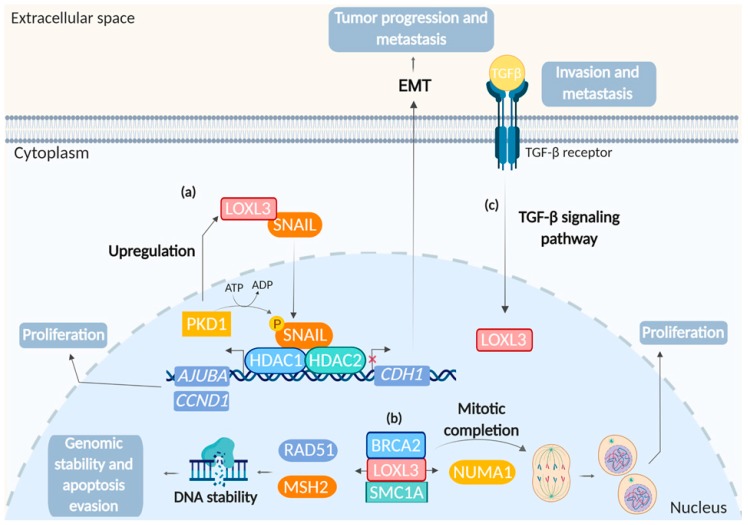
Role of LOXL3 in tumor. (**a**) Tumor progression and metastasis: LOXL3 physically interacts with SNAIL in perinuclear region, preventing the SNAIL degradation and nuclear export. SNAIL represses the *CDH1* (E-cadherin gene) transcription, which induces epithelial-mesenchymal transition (EMT). Protein kinase D1 (PKD1) phosphorylates SNAIL and upregulates LOXL3 expression. The phosphorylation at Ser11 increases the interaction of SNAIL and LOXL3, as well as of SNAIL and HDAC1 and 2. SNAIL and HDACs complex is stabilized in the nucleus and promotes upregulation of proliferation markers, such as *AJUBA* and *CCND1* (cyclin D1 gene). (**b**) Genomic stability and sustained proliferation: LOXL3 interacts with proteins involved in genomic integrity, as BRCA2, SMC1A, NUMA1, RAD51, and MSH2, with consequent DNA repair and evasion of apoptosis. Additionally, interaction of LOXL3 with BRCA2, SMC1A and NUMA1 is important to mitotic completion, which contributes to melanocyte transformation. (**c**) Invasion and metastasis: *LOXL3* expression is downstream from TGF-β signaling pathway in gastric cancer. LOXL3 is essentially localized in the nucleus and the expression correlates with prognosis of patients. Created with BioRender.

LOXL3 plays a role in the repression of E-cadherin gene (*CDH1*) expression through physical interaction with SNAIL (Figure 6a), a transcription factor involved in the epithelial-mesenchymal transition (EMT) process. LOXL3 interaction with SNAIL in the perinuclear envelope cooperates to downregulate *CDH1* expression, thus suggesting LOXL3 involvement in tumor progression and metastasis [18]. Furthermore, SNAIL is regulated by protein kinase D1 (PKD1) through phosphorylation at Ser11, and PKD1 promotes the upregulation of LOXL3 and its interaction with SNAIL (Figure 6a). SNAIL phosphorylation at Ser11 results to the binding to histone deacetylases 1 and 2 (HDAC1 and 2), as well as to LOXL3. The complex formed by Snail phosphorylated and HDAC1 and 2 is stabilized in the nucleus, and promotes upregulation of proliferation markers, such as cyclin D1 and AJUBA (Figure 6a) [40]. In melanoma, LOXL3 is involved in tumorigenesis and tumor progression. The upregulation of LOXL3 was associated with the oncogenic BRAF pathway in melanocyte transformation [19]. Additionally, LOXL3 upregulation in melanoma was correlated with LOXL3 promoter hypomethylation. LOXL3 silencing promoted DNA damage response in melanoma cells, with accumulation of double-strand breaks, aberrant mitosis with accumulation of cells in the G2/M phase, and apoptosis. LOXL3 binds to different proteins involved in the maintenance of genome integrity and/or mitosis, such as BRCA2, RAD51, SMC1A, MSH2, SMC3, and NUMA1.

These data support a pro-oncogenic role of LOXL3 in genomic stability and mitotic completion in melanocyte transformation and melanoma survival and progression [19].

In a large cohort of 597 primary gastric carcinoma cases, Kasashima et al. evaluated the expression of LOXL1, LOXL2, and LOXL3 by immunohistochemistry [20]. LOXL3 expression was detected mainly in the nucleus. Positive protein expressions of all three members of LOX family were correlated with tumor invasion, lymph node metastasis, and poorer prognosis of patients [20]. Moreover, TGF-β induced *LOXL3* upregulation in gastric cancer cells, suggesting that LOXL3 was downstream from the TGF-β signaling pathway (Figure 6c) [20].

*LOXL3* expression was detected in breast cancer, both in primary and pleural effusion [41]. In a recent study, LOX, LOXL1, LOXL2, and LOXL3 expressions were evaluated by immunohistochemistry in 291 cases of breast cancer. LOXL3 expression was positive in only 13.4% of cases and correlated with intratumoral and peritumoral inflammation, as well as with expression of estrogen and progesterone receptors and molecular subtypes [42].

LOXL3 expression was also detected in various kinds of tumors, such as myeloproliferative neoplasms [43] and ovarian carcinoma (primary tumor, metastasis, and peritoneum and pleura effusions) [41]. LOXL3 peptide was detected in the plasma of ovarian cancer patients [44]. Furthermore, LOXL3 expression has been detected in circulating tumor cells of colorectal cancer, and its expression has been correlated with treatment response and prognosis [45,46].

## 5. Concluding Remarks

Recent studies on LOXL3 have uncovered novel and diversified roles beyond its canonical function as amino oxidase. Interestingly, LOXL3 plays roles in embryonic development and in the pathogenesis of several diseases, including collagenopathies, fibrosis, and cancer, which highlights LOXL3 as a potential target for therapy. Currently, non-selective pan inhibitor of the LOX family, β-APN, is available. The structural similarities among LOX family members and lack of precise knowledge about their crystal structures make the development of a specific-LOX-member inhibitor a challenge. Nonetheless, a recent report of an LOXL2/LOXL3 dual inhibitor paves the way to obtaining a specific LOXL3 inhibitor for therapeutic purposes [47,48]. A specific LOXL3 inhibitor could be used for the treatment of diverse pathologies. This revision of the most recent works on LOXL3 may motivate and speed up to achievement of this goal.

## Abbreviations

AJUBA	Ajuba LIM protein
AP-1	Activating protein-1
AP-2	Activating protein-2
BMP-1	Bone morphogenetic protein 1
BRAF	B-Raf proto-oncogene
BRCA2	Breast cancer 2
CAAT	CCAAT sequence
*CCND1*	Cyclin D1 gene
*CDH1*	Cadherin-1 gene
*COL2A1*	Collagen type II alpha 1 chain gene
*COL11A1*	Collagen type XI alpha 1 chain gene
CRE	cAMP response element
c-Rel	Proto-oncogene c-Rel
CRL	Cytokine receptor-like
ECM	Extracellular matrix
FGF	Fibroblast growth factor 1
EMT	Epithelial-mesenchymal transition
EST	Expressed sequence tag
FMT	Fibroblast-to-myofibroblast transition
GAGA	GAGA sequence
GATA	GATA sequence
GTEx	Genotype Tissue Expression
HDAC	Histone deacetylase
hTERT	Human telomerase reverse transcriptase
IPF	Idiopathic pulmonary fibrosis
IRF	Interferon regulatory factors
JNK	c-Jun N-terminal kinases
LOX	Lysyl oxidase
LOXL1	Lysyl oxidase-like 1
LOXL2	Lysyl oxidase-like 2
LOXL3	Lysyl oxidase-like 3
LOXL4	Lysyl oxidase-like 4
LTQ	Lysyl tyrosyl quinone
MIBP	Muscle integrin binding protein
MSH2	MutS homolog 2
NF-κB	Nuclear factor kappa B
NRSF	Neural restrictive silencer factor
NUDR	Nuclear DEAF-1-related
NUMA1	Nuclear mitotic apparatus protein 1
PAX	Paired box
PDGF	Platelet-derived growth factor
PKD1	Protein kinase D1
Pit1	Pituitary transcript factor 1
RAD51	DNA repair protein RAD51 homolog 1
RFX1	Regulatory factor X1
SRCR	Scavenger receptor cysteine-rich
SNAIL	Snail homolog 1
SMC1A	Structural maintenance of chromosomes 1A
SMC3	Structural maintenance of chromosomes 3
SRF	Serum response element
SS	Stickler Syndrome
STAT3	Signal transducer and activator of transcription 3
TATA	TATAAA sequence
TGF	Transforming growth factor
TPM	Transcripts per million
UTR	Untranslated region
YY1	Yin yang 1
β-APN	β-aminopropionitrile
